# The Interplay Between Immunometabolism and Neuroinflammation in Alzheimer’s Disease

**DOI:** 10.3390/biom16050656

**Published:** 2026-04-28

**Authors:** Tiziana Di Crescenzo, Giulio Papiri, Valentina Membrino, Sonila Alia, Monia Cecati, Roberto Campagna, Mauro Silvestrini, Simona Luzzi, Arianna Vignini

**Affiliations:** 1 Department of Clinical Sciences, Section of Biochemistry, Biology and Physics, Università Politecnica delle Marche, 60126 Ancona, Italy; t.dicrescenzo@pm.univpm.it (T.D.C.); v.membrino@pm.univpm.it (V.M.); s.alia@staff.univpm.it (S.A.); 2 Neurology Unit, Ospedale Provinciale ‘Madonna del Soccorso’, 63074 San Benedetto del Tronto, Italy; giulio.papiri@outlook.it; 3 Department for the Promotion of Human Science and Quality of Life, San Raffaele Roma University, 00166 Rome, Italy; monia.cecati@uniroma5.it (M.C.); roberto.campagna@uniroma5.it (R.C.); 4 Department of Experimental and Clinical Medicine, Università Politecnica delle Marche, 60126 Ancona, Italy; m.silvestrini@staff.univpm.it (M.S.); s.luzzi@staff.univpm.it (S.L.); 5 Neurological Unit, Azienda Ospedaliero Universitaria delle Marche, 60126 Ancona, Italy

**Keywords:** Alzheimer’s disease, neuroinflammation, immunometabolism, microglia, polyphenols, AMPK, SIRT1, NLRP3, mitochondria

## Abstract

Alzheimer’s disease (AD) is a multifactorial neurodegenerative disorder characterized by progressive cognitive decline and neuropathological hallmarks such as amyloid-β plaques and neurofibrillary tangles. In recent years, chronic neuroinflammation has emerged as a central mechanism linking genetic, metabolic, and immune dysfunctions in AD. Activated microglia and astrocytes release pro-inflammatory cytokines and reactive oxygen species that exacerbate synaptic and neuronal injury, while impaired clearance mechanisms and blood–brain barrier disruption further sustain inflammation. A growing body of research highlights the role of immunometabolism—the bidirectional interaction between immune activation and cellular metabolism—in shaping glial phenotypes and disease progression. Dysregulation of glucose, lipid, and amino acid metabolism, together with alterations in key metabolites such as lactate, NAD^+^, and reactive oxygen species, promotes a maladaptive inflammatory state. Genetic factors including APOE4 and TREM2 variants affect microglial lipid handling pathways, while systemic metabolic disorders and gut microbiota alterations amplify neuroinflammatory cascades. Natural bioactive compounds, particularly polyphenols, have gained attention for their ability to modulate immunometabolic pathways. By activating AMPK and SIRT1 and inhibiting mTOR and NLRP3 inflammasome signaling, polyphenols may tune mitochondrial function, redox homeostasis, and autophagy, promoting adaptation to chronic metabolic stress. Therefore, metabolic-immune interactions represent pleiotropic therapeutic avenues for AD. Understanding how immunometabolites and nutrient-sensing pathways regulate compartmentalized inflammation in the CNS may pave the way for novel interventions that combine metabolic precision with neuroprotective efficacy.

## 1. Introduction

Alzheimer’s disease (AD) is a progressive neurodegenerative disorder characterized by cognitive decline mainly beginning with memory loss and cognitive-behavioural impairment, occurring within the continuum of a progressive proteinopathy, characterized by accumulation of senile plaques, including various amyloid-β (Aβ) isoforms, and neurofibrillary tangles, composed of hyperphosphorylated tau. Despite decades of research, effective disease-modifying therapies remain elusive.

AD is increasingly recognized as a multifactorial disorder involving not only amyloid-β and tau pathology but also neuroimmune dysregulation, metabolic dysfunction, and systemic factors [1,2].

The classical amyloid cascade hypothesis has evolved into a more integrative model in which amyloid pathology interacts with immune and metabolic dysfunctions, with immunometabolism emerging as a key interface linking cellular energy status to inflammatory responses.

Under a chronological perspective, AD pathogenesis has been theorized to begin with a biochemical phase, where the disease process is mainly confined within neurons, followed by a cellular phase, where small glioneuronal cellular networks become dysfunctional, igniting amyloid/tau spreading and inflammation, followed by a clinical phase, where these processes, becoming widespread, disrupt large-scale networks. During the cellular and clinical phase, inflammatory responses occurring within the central nervous system (CNS) are tightly regulated by a crosstalk between inflammatory and metabolic pathways, which is influenced, to a certain degree, by bidirectional signals produced by the Immune system and the gut–brain axis.

While the CNS is traditionally considered immune-privileged, increasing evidence supports dynamic interactions with peripheral immune cells, particularly within meningeal and perivascular compartments, although the extent of parenchymal infiltration under physiological conditions remains debated [1,3].

Aberrant inflammation is being increasingly recognized as an early process which might be influenced, on a local and systemic scale, by degeneration of discrete areas of the CNS. Degeneration of the nucleus of tractus solitarius (NTS), a brainstem integration nucleus for visceral sensibility, is an early event of streptozotocin AD mice models, and results in upregulation of systemic inflammatory responses. This pathway may represent an additional layer of systemic immunometabolic regulation, linking autonomic control of peripheral inflammation with central neuroimmune responses. Intriguingly, NTS dopaminergic neurons appear as a pivotal anti-inflammatory effector pathway [4,5]. In line with these findings, degeneration of brainstem monoaminergic projection systems, such as the noradrenergic locus coeruleus (LC) and the dopaminergic ventral tegmental area, which might occur before amyloid and tau pathology spreading, has been shown to affect nerve cell loss and aberrant immune/inflammatory responses within the CNS [6], through bidirectional context-dependent effects on innate and adaptive immune responses, promoting either proinflammatory or tolerogenic cell responses [7]. Altered monoaminergic signaling might not be only confined to the CNS, since peripheral effector cells might modify their responses to catecholamines; in fact it has been shown that AD lymphocytes display a reduced expression of D2-like receptors, suggesting altered responses to dopamine [8]. Circulating lymphocytes of AD patients have also been found to display increased sensitivity to β-adrenergic agonists [9]. In addition, β-adrenergic receptor activation in microglia has been shown to downregulate proinflammatory pathways, such as proinflammatory cytokine secretion, major histocompatibility complex class II (MHCII) and nitric oxide synthase type 2 (NOS2) expression [10]. On the whole, dysregulation of monoaminergic signalling is thought to give an early important contribution to the AD pathogenesis, affecting peripheral effectors and glial cells.

Recent studies have identified distinct microglial activation states, including disease-associated microglia (DAM), which exhibit specific metabolic and transcriptional signatures linked to lipid metabolism and phagocytic activity [11].

Microglia and astrocytes become chronically activated in response to aggregated Aβ and tau, releasing pro-inflammatory cytokines such as interleukin-1β (IL-1β), tumor necrosis factor-α (TNF-α), and interleukin-6 (IL-6), along with reactive oxygen (ROS) and nitrogen species (RNS). These mediators exacerbate synaptic and neuronal dysfunction [12]. Persistent inflammation not only contributes to neuronal death but also impairs clearance mechanisms, including microglial phagocytosis, glymphatic outflow and blood–brain barrier (BBB) integrity, thereby amplifying amyloid deposition and tau propagation while also enabling incremental infiltration and activation of T lymphocytes from peripheral tissues, feeding a self-sustaining loop [13,14,15].

Notably, systemic inflammation, arising from infection, metabolic disease, or ageing (“inflammaging”), might accelerate these processes, promoting further damage [16]. While acute inflammatory responses can facilitate Aβ clearance and tissue repair, persistent inflammation might advance signaling pathways disrupting CNS energy production, advancing loss of cellular and synaptic elements, and enabling synaptic/large-scale network dysfunction, which underlie cognitive decline [17].

Genetic evidence reinforce this link: several risk loci for sporadic AD encode immune-modulating genes, suggesting that dysregulated immune processes are not merely downstream consequences but drivers of pathology [13]. Increasing attention has turned to immunometabolites, molecules that bridge immune signaling and metabolism, as key modulators of inflammation in AD. Natural compounds, particularly polyphenols abundant in Mediterranean-style diets, may influence these pathways.

Given this expanding evidence, therapeutic strategies targeting neuroinflammation, through cytokine modulation, inflammasome inhibition, or restoration of glial homeostasis, show promise especially if implemented early and selectively to avoid impairing protective immune functions.

In this context, the present review aims to provide an integrative perspective on the interplay between immunometabolism and neuroinflammation in AD. Specifically, we focus on how metabolic rewiring of microglia and astrocytes regulates inflammatory signaling and how key immunometabolites act as molecular mediators between metabolic stress and immune activation. Furthermore, we discuss emerging evidence that dietary bioactive compounds, particularly polyphenols, may modulate these pathways by targeting nutrient-sensing signaling networks and inflammasome activation. By integrating metabolic, genetic, and immunological perspectives, this review highlights immunometabolism as a central axis in AD pathogenesis and a potential target for therapeutic intervention.

## 2. Neuroinflammation and Immunometabolism in Alzheimer’s Disease

### Neuroinflammation in AD: The Immune Players

Microglia, the resident immune cells of the CNS β, are pivotal in AD-associated inflammation. In response to Aβ and tau pathology, they secrete cytokines (IL-1β, TNF-α, IL-6) and activate inflammasome pathways such as NLRP3, exacerbating neuronal injury. Chronic activation promotes dysfunctional, neurotoxic phenotypes. Astrocytes, perivascular macrophages, and infiltrating peripheral immune cells reinforce this inflammatory environment. Inflammation in the CNS is tightly linked to metabolic state: immune cells must adjust energy production to sustain their effector functions. The emerging field of immunometabolism—the interplay between immune activity and cellular metabolism—has become essential to understanding chronic neuroinflammation [18]. Microglia exhibit high metabolic flexibility, alternating between glycolysis, oxidative phosphorylation (OXPHOS), and alternative pathways depending on environmental cues, which enables them to assume a spectrum of intermediate phenotypes, such as disease-associated microglia, interferon-responsive microglia, activated response microglia, terminally inflammatory microglia, triggering receptor expressed on myeloid cells 2 (TREM2+) senescent microglia, whose properties are reviewed in depth elsewhere [19].

In AD, maladaptive metabolic reprogramming impairs phagocytosis and clearance of Aβ and tau [20]. Disruption of metabolic-immune homeostasis may underlie the susceptibility of the aging brain to neurodegeneration [21]. Experimental evidence suggests that overstimulation of inflammatory pathways might represent an early feature in AD, producing detrimental effects on synaptic functioning and remodeling: a recent cross-sectional biomarker study has detected a positive correlation between increasing IL6 concentration in the CSF and markers of neuroaxonal loss, such as NfL, suggesting that chronic inflammation might hasten the progression of neurodegenerative processes [22]. In addition, in vitro evidence, as well as data on MS patients, suggest that elevated IL-6 negatively affects LTP-like responses, thus reducing brain/synaptic plasticity, therefore favouring maladaptative responses to the neurodegenerative process, hypothetically reducing the so-called “cognitive reserve” [23].

On the other hand, persistent stimulation of inflammatory pathways might hasten the transition of lymphoid and myeloid cells involved in CNS homeostasis, towards a senescent “immune-exhausted” phenotype. At variance from animal models, data from unimpaired subjects shows that increased Interferon-Gamma production might be associated to slower cognitive decline [24], while AD patients display reduced T-lymphocyte interferon gamma (IFN-γ) binding [25], suggesting that diminished IFN-γ signalling might accompany the transition between the presymptomatic and symptomatic phases, perhaps reflecting immune ageing [19].

## 3. Key Immunometabolic Pathways and Metabolites in AD

In the following section, we provide an overview of the major metabolic pathways that play a key role in AD and discuss how alterations in these metabolic axes influence the behavior, function, and activation states of immune cells involved in the disease process.

A key regulator of the metabolic shift toward glycolysis in activated immune cells is hypoxia-inducible factor-1α (HIF-1α). Under inflammatory or hypoxic conditions, HIF-1α stabilization promotes transcription of glycolytic enzymes, including hexokinase-2 (HK2) and pyruvate kinase M2, thereby enhancing glycolytic flux and supporting the energetic requirements of activated microglia. Importantly, HIF-1α signaling is also linked to inflammatory pathways, as it can directly promote transcription of pro-inflammatory cytokines such as IL-1β.

In contrast, the AMP-activated protein kinase (AMPK) pathway acts as a metabolic sensor that promotes energy homeostasis. Activation of AMPK inhibits the mechanistic target of rapamycin (mTOR), thereby promoting autophagy and mitochondrial quality control. In microglia, AMPK activation has been associated with anti-inflammatory phenotypes and improved clearance of protein aggregates. Conversely, chronic activation of mTOR signaling suppresses autophagy and contributes to the accumulation of dysfunctional mitochondria and misfolded proteins.

**Figure 1:**
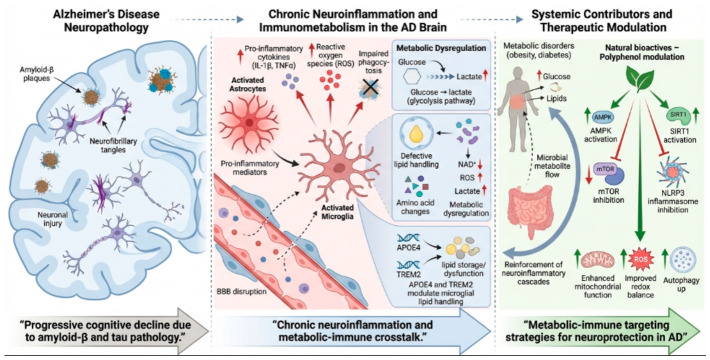
It illustrates the interplay between core Alzheimer’s disease pathologies (amyloid-β plaques, neurofibrillary tangles) and chronic neuroinflammation driven by activated astrocytes and microglia. Metabolic dysregulation, involving glucose and lipid handling (influenced by factors like APOE4/TREM2), reinforces these neuroinflammatory cascades. Systemic contributors such as metabolic disorders (obesity, diabetes) exacerbate the condition. Potential therapeutic strategies include modulation via natural bioactives (polyphenols) targeting pathways like AMPK, SIRT1, and mTOR to enhance mitochondrial function and restore redox balance, aiming for neuroprotection.

These metabolic sensors intersect with innate immune signaling through activation of the NLRP3 inflammasome, a multiprotein complex responsible for the maturation of IL-1β and IL-18. Metabolic stress, mitochondrial dysfunction, and accumulation of danger-associated molecular patterns can all trigger NLRP3 activation, thereby linking metabolic dysregulation to neuroinflammatory amplification (Figure 1).

### 3.1. Glucose Metabolism and Glycolysis/OXPHOS Balance

Activation of microglia is typically associated with a metabolic shift toward glycolysis, similar to the Warburg effect, which supports rapid energy production and inflammatory responses. Rather than impairing glucose metabolism, stimuli such as Aβ or lipopolysaccharide (LPS) enhance glycolytic flux while often reducing oxidative phosphorylation efficiency.

Chronic reliance on glycolysis, however, depletes energy reserves and impairs mitochondrial function, potentially contributing to dysfunctional local innate immune responses [26]. Mitochondrial dysfunction represents a common pathway in neurodegenerative processes and a key feature of AD pathogenesis; both Aβ and the glucose-lowering hormone amylin, which constitute the main components of senile plaques, have been shown to exert a toxic effect on mitochondria, reducing ATP production [27]. Mitochondrial dysfunction ultimately links disrupted ATP production to impaired ion homeostasis, excessive ROS production and endoplasmic reticulum (ER) stress, giving rise to a self-sustaining loop of cytotoxicity and inflammation, while also impairing the brain’s ability to fuel its housekeeping processes via glucose oxidation [28]. Glucose metabolism impairment in AD is complex and occurs on several levels; glyceraldehyde, which is considered one of the main precursors of advanced glycation end products (AGEs) [29], has been found, in cell models, to act as a “metabolic toxin”, partly by inducing amyloidogenic APP processing, but also by disrupting glycolytic and mitochondrial metabolism, resulting in detrimental changes to redox balance and calcium homeostasis [30].

These processes have been regarded as key drivers of AD pathogenesis since they occur in AD-vulnerable brain regions ahead of amyloid or tau deposition, while also impacting inflammatory and immune responses [31]. In fact, peripheral lymphocytes from AD and mild cognitive impairment (MCI) patients display deregulated cell cycle entry and metabolic reprogramming pathways, sustained by overactivation of the PI3K/AKT/mTOR pathway, leading to enhanced proliferation, which relies on aerobic glycolysis [32]. In addition, HIF-1α promotes this glycolytic shift by inducing enzymes such as HK2. In AD microglia, upregulation of HIF-1α enhances glycolysis at the expense of mitochondrial respiration [33]. The pentose phosphate pathway (PPP), which supplies NADPH and ribose-5-phosphate, is also impaired in AD, weakening antioxidant defenses.

#### Mitochondrial Dysfunction and Immunometabolism

Mitochondrial dysfunction represents a central hub linking metabolic failure and inflammatory signaling in AD. Impaired mitochondrial activity leads to defective oxidative phosphorylation, reduced ATP production, and increased electron leakage from the respiratory chain, resulting in excessive generation of mitochondrial reactive oxygen species (mtROS). These mtROS act as potent danger-associated molecular patterns (DAMPs) that can trigger activation of the NLRP3 inflammasome, thereby promoting caspase-1 activation and the release of pro-inflammatory cytokines such as (IL-1β) [34,35].

In parallel, mitochondrial quality control mechanisms are frequently compromised in AD. In particular, impaired mitophagy—the selective autophagic removal of damaged mitochondria—leads to the accumulation of dysfunctional organelles and sustained oxidative stress. Defective mitophagy has been associated with increased inflammasome activation and chronic neuroinflammation, further amplifying neuronal damage [36,37].

Importantly, the consequences of mitochondrial dysfunction differ between neurons and microglia. Neurons rely predominantly on oxidative phosphorylation to sustain their high energetic demands; therefore, mitochondrial impairment rapidly results in bioenergetic failure, synaptic dysfunction, and ultimately cell death [38]. In contrast, microglia exhibit greater metabolic flexibility and can shift toward glycolysis during activation. While this metabolic reprogramming supports rapid immune responses, prolonged mitochondrial damage in microglia promotes a chronically activated phenotype characterized by increased ROS production, impaired phagocytosis, and sustained inflammatory signaling [28,39].

**Figure 2:**
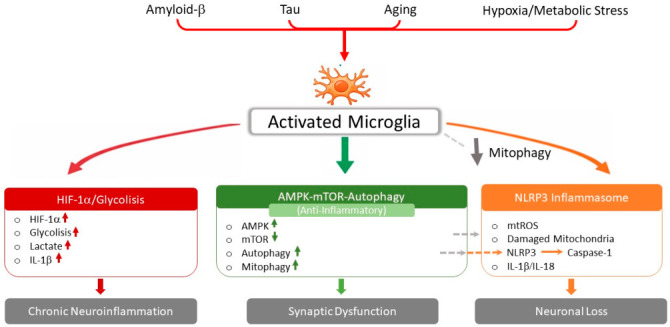
Immunometabolic signaling pathways in Alzheimer’s disease. Amyloid-β aggregation, tau pathology, metabolic stress, and aging promote metabolic rewiring of microglia. Stabilization of HIF-1α enhances glycolysis and pro-inflammatory cytokine production. In parallel, dysregulation of the AMPK–mTOR axis impairs autophagy and mitochondrial quality control. Mitochondrial damage and reactive oxygen species activate the NLRP3 inflammasome, leading to caspase-1 activation and IL-1β release. These interconnected pathways amplify chronic neuroinflammation and contribute to synaptic dysfunction and neuronal degeneration.

Overall, mitochondrial dysfunction not only contributes directly to neuronal degeneration but also acts as a key driver of maladaptive immunometabolic responses in glial cells, reinforcing the vicious cycle between metabolic stress and chronic neuroinflammation in AD (Figure 2).

### 3.2. Lipid Metabolism and Cholesterol Handling

Lipid dysregulation is a hallmark of AD. Microglia often accumulate lipid droplets (LDs), forming lipid-droplet–accumulating microglia (LDAMs) with reduced phagocytic activity and a pro-inflammatory profile [33]. Mutations in TREM2 and certain apolipoprotein E (APOE) isoforms (notably APOE4) disrupt lipid clearance, promoting LD formation and metabolic stress. Impaired lipid handling by phagocytosis is thought to play an important role in late-onset AD pathogenesis; in a recent study, mutations in the phosphatidylinositol-binding clathrin assembly protein (PICALM) gene, which regulates microglial debris clearance, have been associated with a causal role in AD [40]. The signaling pathway PGC-1-PPARγ modulates lipid metabolism and may shift microglia toward a more homeostatic phenotype. Growing evidence suggests that drugs affecting systemic glucose and lipid metabolism might be beneficial on these signaling pathways: glucagon-like peptide-1 (GLP-1) receptor agonists have been found to affect, via a PPARγ-dependent switch, macrophage polarization towards a M2 phenotype and might also affect via direct and indirect mechanisms microglial polarization [41,42]. Lipid dysregulation might take place at lipid rafts, which are considered the main cellular hubs for cholesterol trafficking. Notably, in cerebral cortices from AD patients, lipid rafts have been found to display an altered architecture and composition, characterized by a reduction in polyunsaturated fatty acid content (lower ω-3 PUFA and MUFA) [43,44,45]. In addition, data from biomarker studies, show an increase, in CSF from AD patients, of pro-inflammatory arachidonic acid derivatives, which are to contribute to the disease process [46].

### 3.3. Amino Acid Metabolism and the Kynurenine Pathway

The kynurenine pathway represents a major route of tryptophan metabolism and plays a critical role in neuroinflammation. Its metabolites include neurotoxic compounds such as quinolinic acid, which promotes excitotoxicity through NMDA receptor activation, and neuroprotective molecules such as kynurenic acid. Dysregulation of this pathway has been implicated in both neuronal damage and immune activation in AD.

The kynurenine pathway is activated by inflammatory stimuli through indoleamine 2,3-dioxygenase (IDO). Resulting metabolites such as quinolinic acid are neurotoxic, whereas kynurenic acid can be neuroprotective. Imbalance in this pathway contributes to excitotoxicity and chronic inflammation in AD [33]. Other amino acids (e.g., glutamine, arginine) may also feed into immunometabolic regulation via roles in biosynthesis, mitochondrial anaplerosis, or nitric oxide production.

### 3.4. Succinate and Arginine Metabolism in Immunometabolic Regulation

Among tricarboxylic acid (TCA) cycle intermediates, succinate has emerged as a key immunometabolite linking mitochondrial metabolism to inflammatory signaling. During inflammatory activation, accumulation of succinate stabilizes hypoxia-inducible factor-1α (HIF-1α), which in turn promotes transcription of glycolytic enzymes and pro-inflammatory mediators, including interleukin-1β (IL-1β). This mechanism establishes a direct connection between metabolic rewiring and inflammatory responses and has been extensively characterized in activated macrophages and microglia [47,48]. In addition, extracellular succinate can act through its receptor SUCNR1, further amplifying inflammatory signaling and contributing to immune cell activation.

Arginine metabolism also plays a pivotal role in regulating immune responses in the central nervous system. Arginine can be metabolized by inducible nitric oxide synthase (iNOS) to generate nitric oxide (NO), a reactive molecule associated with pro-inflammatory responses, oxidative stress, and neuronal damage. Alternatively, arginine can be processed by arginase-1 (ARG1) to produce ornithine and polyamines, which are generally linked to tissue repair, anti-inflammatory functions, and resolution of inflammation. The balance between these two metabolic pathways is a critical determinant of microglial polarization and functional phenotype [49,50].

In the context of Alzheimer’s disease, dysregulation of arginine metabolism has been associated with sustained inflammatory activation and impaired tissue repair mechanisms. Increased iNOS expression and nitric oxide production contribute to oxidative damage, while alterations in ARG1 activity may disrupt homeostatic microglial functions. Thus, the interplay between succinate signaling and arginine metabolism further underscores the role of metabolic intermediates as active regulators of neuroinflammation and disease progression.

### 3.5. Other Metabolites: Lactate, ROS, and NAD^+^

Beyond classical metabolic intermediates, several small metabolites—lactate, ROS, and NAD^+^/NADH—have emerged as critical regulators of the immune-metabolic crosstalk in AD. Enhanced glycolysis elevates lactate production, now recognized as a signaling molecule influencing gene expression through histone lactylation. Elevated lactate can modulate glial activity and contribute to neuroinflammation [51,52].

Excessive mitochondrial ROS generation damages DNA, lipids, and proteins, triggering NLRP3 inflammasome activation, perpetuating cytokine release and leading to cell death [53,54].

Declining NAD^+^ levels in aging and AD impair sirtuin-dependent regulation of metabolism and inflammation, further compromising mitochondrial function. cGAS-STING has recently emerged as a critical signaling pathway, affecting both cell stress and inflammatory signaling, which becomes deregulated in NAD^+^ deficiency [55].

Restoring NAD^+^ through precursors such as nicotinamide riboside or nicotinamide mononucleotide ameliorates neuroinflammation and improves cognition in animal models, likely through modulation of sirtuin-dependent pathways and mitochondrial function [56,57]. Together, these metabolites act as active regulators—not mere byproducts—of neuroimmune signaling, forming self-amplifying cycles of metabolic stress and inflammation.

**Figure 3:**
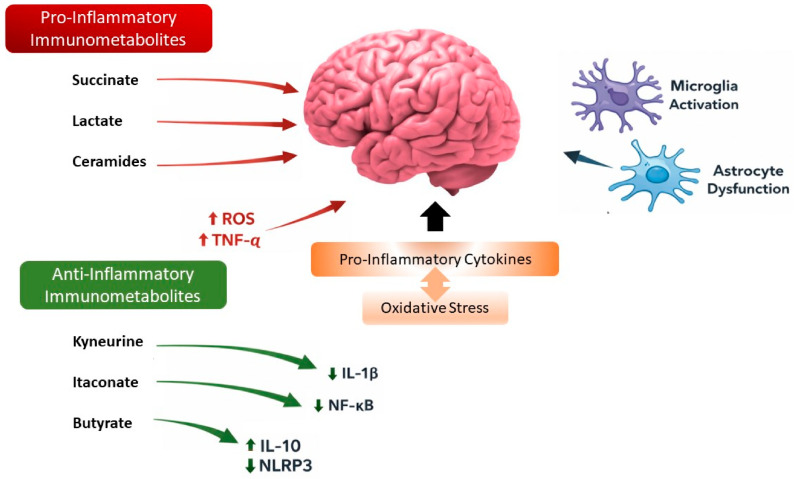
Immunometabolites linking metabolism and neuroinflammation in Alzheimer’s disease. Metabolic intermediates derived from glycolysis, the tricarboxylic acid cycle, and redox metabolism act as signaling molecules regulating immune responses in the brain. Lactate and succinate promote inflammatory pathways through epigenetic regulation and HIF-1α stabilization, whereas NAD^+^ depletion and mitochondrial ROS production amplify oxidative stress and inflammasome activation. Bioactive compounds such as polyphenols may counteract these processes by modulating AMPK, SIRT1, and antioxidant pathways.

In summary, metabolites like lactate, ROS, and NAD^+^ are not passive byproducts of altered metabolism but active modulators of neuroimmune communication. Their dysregulation contributes to a self-reinforcing loop of metabolic stress and inflammation that accelerates neuronal vulnerability in Alzheimer’s disease (Figure 3).

## 4. Genetic and Systemic Factors Bridging Immunometabolism and AD

Genetic studies over the past decade have significantly reshaped the understanding of AD pathogenesis. Genome-wide association studies (GWAS) have identified numerous risk loci that are strongly enriched in genes expressed in microglia, highlighting the central role of immune regulation and metabolic adaptation in disease progression. Many of these genes influence lipid metabolism, phagocytosis, and inflammatory signaling, thereby linking genetic susceptibility to immunometabolic dysregulation within the central nervous system.

Among the most prominent genetic determinants, APOE remains the strongest risk factor for sporadic late-onset AD. The APOE4 isoform is associated with impaired lipid transport, altered cholesterol homeostasis, and enhanced neuroinflammatory responses. In microglia, APOE4 promotes a shift toward glycolytic metabolism and pro-inflammatory activation states, thereby reducing the efficiency of amyloid-β clearance and exacerbating neuronal injury [58,59].

Another key regulator of microglial metabolism and survival is TREM2. TREM2 is a lipid-sensing receptor expressed by microglia that modulates phagocytosis, energy metabolism, and inflammatory responses. Loss-of-function variants of TREM2 impair microglial ability to respond to amyloid plaques and lead to defective lipid handling and mitochondrial dysfunction. Experimental evidence suggests that TREM2 signaling supports metabolic fitness in microglia by promoting oxidative metabolism and lipid utilization, which are essential for sustaining microglial responses during neurodegeneration [39,60].

Beyond APOE and TREM2, several additional AD risk genes contribute to immune regulation. CD33, a member of the sialic acid-binding immunoglobulin-like lectin (SIGLEC) family, negatively regulates microglial phagocytosis. Increased CD33 expression has been associated with reduced amyloid-β clearance and enhanced inflammatory signaling, suggesting that this receptor acts as an inhibitory checkpoint of microglial activity [61]. Similarly, complement receptor 1 (CR1) is involved in complement-mediated immune responses and synaptic pruning. Variants of CR1 have been linked to increased AD risk and may promote complement-driven neuroinflammation and synaptic loss [62].

Another gene strongly associated with AD susceptibility is bridging integrator 1 (BIN1), which participates in membrane trafficking, endocytosis, and cytoskeletal dynamics. Although BIN1 is highly expressed in neurons, recent evidence suggests that it may also influence immune signaling and microglial responses to amyloid pathology. Dysregulation of endocytic processes mediated by BIN1 may contribute to altered amyloid processing and inflammatory signaling within the brain [63].

Recent advances in single-cell transcriptomics have further identified a specific microglial activation state, the DAM. This phenotype emerges in response to neurodegenerative stimuli and is characterized by the upregulation of genes involved in lipid metabolism, phagocytosis, and inflammatory signaling. Transition to the DAM state occurs in a two-step process that is partly dependent on TREM2 signaling and involves the coordinated regulation of genes such as APOE, LPL, and CST7 [64]. Importantly, the DAM program reflects profound metabolic reprogramming of microglia, including enhanced lipid metabolism and altered mitochondrial function, further supporting the concept that metabolic pathways are tightly linked to immune responses in neurodegeneration.

Collectively, these genetic findings indicate that many AD risk genes converge on pathways controlling microglial metabolism, lipid handling, and inflammatory signaling. This convergence reinforces the concept that AD is not only a disorder of protein aggregation but also a disease driven by dysregulated immunometabolic networks within the brain.

## 5. Polyphenols as Modulators of Neuroinflammation and Immunometabolism in Alzheimer’s Disease

Immunometabolites such as lactate, succinate, and itaconate have emerged as key regulators of immune cell function, linking metabolic pathways to inflammatory responses in the central nervous system. These metabolites can modulate the activation state of microglia, astrocytes, and peripheral immune cells, promoting either pro-inflammatory or anti-inflammatory phenotypes depending on the metabolic context [65,66]. In particular, succinate accumulation stabilizes HIF-1α, thereby enhancing glycolysis and driving the production of pro-inflammatory cytokines such as IL-1β, whereas other metabolites may exert regulatory or compensatory effects on immune signaling.

In this context, dietary polyphenols have attracted increasing attention as modulators of immunometabolic pathways in neurodegenerative diseases. Beyond their well-established antioxidant properties, polyphenols exert pleiotropic regulatory effects on cellular signaling networks involved in energy metabolism, mitochondrial function, and inflammatory responses. A growing body of evidence indicates that these compounds can influence key immunometabolic regulators, including AMPK, SIRT1, Nrf2, and the NLRP3 inflammasome, thereby modulating microglial activation and neuroinflammatory processes [67,68].

Polyphenols such as resveratrol and quercetin have been shown to activate the AMPK/SIRT1 axis, promoting mitochondrial biogenesis, enhancing oxidative phosphorylation, and restoring redox balance.

This metabolic shift counteracts the glycolytic and pro-inflammatory phenotype typically observed in activated microglia. In parallel, inhibition of the mammalian mTOR pathway promotes autophagy and mitophagy, facilitating the clearance of damaged mitochondria and reducing the accumulation of ROS. These effects collectively contribute to the suppression of chronic inflammatory signaling [68,69,70].

Although most evidence derives from preclinical studies, polyphenols represent promising modulators of immunometabolic pathways in AD.

Consistently, several studies have demonstrated that polyphenols can inhibit activation of the NLRP3 inflammasome through autophagy-dependent mechanisms. For instance, lychee seed polyphenols have been shown to suppress amyloid-β-induced NLRP3 activation via the AMPK/mTOR/ULK1 pathway, thereby preserving blood–brain barrier integrity and reducing neuroinflammation in APP/PS1 models [71,72]. Similarly, polyphenol-derived metabolites such as dihydro-resveratrol can attenuate inflammasome activation by promoting mitophagy and improving mitochondrial quality control [73].

Mitochondria represent a critical interface between metabolism and inflammation, and their dysfunction is a hallmark of AD pathogenesis. Polyphenols have been shown to enhance mitochondrial respiration and stimulate peroxisome proliferator-activated receptor gamma coactivator 1-alpha (PGC-1α)-dependent mitochondrial biogenesis, while simultaneously activating Nrf2-mediated antioxidant defenses. In addition, these compounds promote the autophagic removal of damaged organelles, thereby limiting oxidative stress and preventing inflammasome activation [67,74].

Beyond their metabolic effects, polyphenols can also modulate the epigenetic landscape of immune cells. By influencing histone acetylation and DNA methylation through regulation of histone deacetylases (HDACs) and acetyltransferases, polyphenols contribute to the establishment of long-lasting anti-inflammatory phenotypes and increased cellular resilience to stress [68]. This epigenetic–metabolic crosstalk further supports the concept that dietary compounds can induce durable changes in immune cell function.

Overall, these findings highlight the dual role of immunometabolites and polyphenols in shaping neuroinflammatory responses in AD. While endogenous metabolites act as signaling molecules that can either promote or resolve inflammation, dietary polyphenols appear to counteract maladaptive immunometabolic reprogramming by restoring mitochondrial function, reducing oxidative stress, and suppressing inflammasome activation. These properties position polyphenols as promising modulators of the metabolic–inflammatory axis in neurodegeneration.

A summary of the most studied polyphenols and their mechanisms of action in experimental AD models is reported in Table 1.

## 6. Knowledge Gaps and Future Directions

Despite growing interest in immunometabolism in neurodegeneration, several important questions remain unresolved. First, the temporal relationship between metabolic dysfunction and inflammatory activation in AD is still unclear. It remains to be determined whether metabolic alterations represent an early driver of microglial activation or rather a downstream consequence of amyloid-β and tau pathology. Emerging evidence suggests that metabolic reprogramming may precede overt neurodegeneration, but causality has not yet been definitively established [1].

Second, the heterogeneity of microglial metabolic states across different stages of the disease requires further investigation. Recent advances in single-cell transcriptomics have revealed the existence of multiple microglial phenotypes, DAM, which exhibit distinct metabolic and functional signatures [11]. However, the metabolic pathways underpinning these phenotypic transitions remain incompletely understood. Integration of single-cell transcriptomic, proteomic, and metabolomic approaches will be crucial to elucidate how metabolic programs evolve during disease progression and to identify stage-specific therapeutic targets [33].

Another major challenge lies in translating immunometabolic insights into effective clinical interventions. Although several compounds targeting metabolic pathways—such as AMPK activators, NAD^+^ precursors, and mitochondrial modulators—have shown promising results in preclinical models, their efficacy and safety in human patients remain to be fully established. For instance, modulation of microglial metabolism may have context-dependent effects, potentially impairing beneficial immune responses if not carefully controlled [2,31].

Finally, the identification of reliable biomarkers reflecting immunometabolic alterations represents a critical unmet need. Future studies integrating metabolic biomarkers, advanced neuroimaging techniques, and longitudinal clinical data will be essential to determine whether targeting immunometabolism can effectively modify disease progression. A better understanding of the systemic dimension of AD, including the contribution of peripheral metabolism and the gut–brain axis, may further open new avenues for therapeutic intervention [84].

## 7. Conclusions

Growing evidence indicates that AD is not solely a disorder of protein misfolding, but rather a complex interplay between neuroinflammation, immune dysregulation, and metabolic dysfunction. Microglia and astrocytes undergo profound immunometabolic reprogramming that critically influences their capacity to clear pathological aggregates and maintain neuronal homeostasis. Disturbances in glucose, lipid, and amino acid metabolism—together with redox imbalance and NAD^+^ depletion—contribute to a self-perpetuating cycle of oxidative stress and chronic inflammation.

Genetic factors, including APOE4 and TREM2 variants, further disrupt metabolic homeostasis and microglial function, while systemic metabolic disorders and gut–brain axis alterations amplify neuroimmune activation. Within this multifactorial framework, immunometabolism emerges as a central hub integrating metabolic and inflammatory signals in AD pathogenesis. Targeting immunometabolic pathways therefore represents a promising therapeutic strategy. In this context, natural polyphenols may act as modulators of key signaling pathways, including AMPK–mTOR–SIRT1, mitochondrial function, and epigenetic regulation. However, most of the current evidence remains preclinical, and further studies are required to establish their efficacy and translational relevance in human disease.

Despite significant advances, several critical questions remain unresolved, including the temporal relationship between metabolic dysfunction and neuroinflammation, the heterogeneity of microglial metabolic states, and the identification of reliable immunometabolic biomarkers. Future research should focus on integrating multi-omics approaches, longitudinal studies, and well-designed clinical trials to determine whether targeting immunometabolism can effectively modify disease progression, and promote cognitive resilience.

## Figures and Tables

**Table 1:** Polyphenols with reported neuroprotective and immunometabolic effects in Alzheimer’s disease models.

Polyphenol	Molecular Targets/Pathways	Experimental Evidence	Reported Effects in AD Models	References
Resveratrol	SIRT1 activation, AMPK signaling, NF-κB inhibition	Cell and animal models; small clinical studies	Reduces neuroinflammation, promotes mitochondrial biogenesis, improves cognitive performance	[69,75]
Curcumin	NF-κB inhibition, NLRP3 inflammasome suppression, antioxidant activity	Cell and animal models	Reduces amyloid aggregation, attenuates microglial activation and oxidative stress	[76]
Epigallocatechin-3-gallate (EGCG)	Nrf2 activation, MAPK signaling, anti-amyloid activity	Cell and animal models	Protects neurons against Aβ toxicity and reduces inflammatory cytokines	[77]
Quercetin	Nrf2 activation, NF-κB inhibition, antioxidant pathways	Animal models	Reduces oxidative stress and microglial activation; improves cognitive function	[70]
Luteolin	JNK inhibition, NF-κB suppression	Animal models	Reduces microglial inflammatory responses and improves memory	[78]
Kaempferol	PI3K/Akt signaling, antioxidant enzymes	Cell and animal models	Anti-inflammatory and neuroprotective effects in neurodegenerative models	[79]
Ferulic acid	ROS scavenging, Nrf2 activation	Animal models	Reduces Aβ deposition and oxidative stress	[80]
Anthocyanins	Antioxidant activity, anti-inflammatory signaling	Animal models and human dietary studies	Improve neuronal signaling and cognitive function	[81]
Oleuropein	Anti-amyloid activity, antioxidant pathways	Cell and animal models	Inhibits amyloid aggregation and reduces oxidative damage	[82]
Genistein	Estrogen receptor signaling, antioxidant activity	Animal models	Reduces amyloid accumulation and improves synaptic function	[83]
Dihydro-resveratrol	Mitophagy activation, NLRP3 inhibition	Animal models	Reduces neuroinflammation via mitochondrial quality control	[73]

## Data Availability

No new data were created or analyzed in this study.
